# Derivation and Characterization of Isogenic *OPA1* Mutant and Control Human Pluripotent Stem Cell Lines

**DOI:** 10.3390/cells14020137

**Published:** 2025-01-17

**Authors:** Katherine A. Pohl, Xiangmei Zhang, Johnny Jeonghyun Ji, Linsey Stiles, Alfredo A. Sadun, Xian-Jie Yang

**Affiliations:** 1 Jules Stein Eye Institute, Department of Ophthalmology, David Geffen School of Medicine, University of California, Los Angeles, CA 90095, USA; kdpohl@g.ucla.edu (K.A.P.); xmzhang@jsei.ucla.edu (X.Z.);; 2 Molecular Biology Institute, University of California, Los Angeles, CA 90095, USA; 3 Department of Molecular and Medical Pharmacology, Davide Geffen School of Medicine, University of California, Los Angeles, CA 90095, USA; 4 Doheny Eye Center, Department of Ophthalmology, University of California, Los Angeles, CA 91103, USA; alfredo.sadun@gmail.com

**Keywords:** *OPA1* gene, dominant optic atrophy, CRISPR-Cas9 editing, isogenic human pluripotent stem cell lines, mitochondria

## Abstract

Dominant optic atrophy (DOA) is the most commonly inherited optic neuropathy. The majority of DOA is caused by mutations in the *OPA1* gene, which encodes a dynamin-related GTPase located to the mitochondrion. OPA1 has been shown to regulate mitochondrial dynamics and promote fusion. Within the mitochondrion, proteolytically processed OPA1 proteins form complexes to maintain membrane integrity and the respiratory chain complexity. Although *OPA1* is broadly expressed, human *OPA1* mutations predominantly affect retinal ganglion cells (RGCs) that are responsible for transmitting visual information from the retina to the brain. Due to the scarcity of human RGCs, DOA has not been studied in depth using the disease affected neurons. To enable studies of DOA using stem-cell-derived human RGCs, we performed CRISPR-Cas9 gene editing to generate *OPA1* mutant pluripotent stem cell (PSC) lines with corresponding isogenic controls. CRISPR-Cas9 gene editing yielded both *OPA1* homozygous and heterozygous mutant ESC lines from a parental control ESC line. In addition, CRISPR-mediated homology-directed repair (HDR) successfully corrected the *OPA1* mutation in a DOA patient’s iPSCs. In comparison to the isogenic controls, the heterozygous mutant PSCs expressed the same OPA1 protein isoforms but at reduced levels; whereas the homozygous mutant PSCs showed a loss of OPA1 protein and altered mitochondrial morphology. Furthermore, *OPA1* mutant PSCs exhibited reduced rates of oxygen consumption and ATP production associated with mitochondria. These isogenic PSC lines will be valuable tools for establishing *OPA1*-DOA disease models in vitro and developing treatments for mitochondrial deficiency associated neurodegeneration.

## 1. Introduction

Dominant optic atrophy (DOA) is the most common inherited optic neuropathy worldwide [1,2]. The disease prevalence is 1:25,000–1:35,000 in most populations, but it can be as high as 1:10,000 in areas with an established founder effect [3,4]. The visual impairment of DOA usually begins in the first two decades of life due to the loss of retinal ganglion cells (RGCs) [3,5]. RGCs are the essential projection neurons that extend axons through the optic nerve to transmit visual signals from the retina to the brain. The majority of DOA is caused by mutations in the gene optic atrophy 1 (*OPA1*; OMIM:*605290), which encodes a dynamin-related GTPase located to the mitochondrion [6,7,8]. Although *OPA1* is broadly expressed by somatic tissues, most cases of DOA are non-syndromic and patients only exhibit symptoms related to RGC degeneration—namely progressive, bilateral vision loss, including reduced visual acuity, color vision defects, and central visual field defects [3,9,10,11]. A hallmark of DOA aiding in its diagnosis is temporal optic nerve head pallor, which is attributed to the preferential loss of RGCs in the papillomacular bundle [12,13]. Although inherited in an autosomal dominant manner, *OPA1* mutations are only ~43-88% penetrant, leading to a high degree of heterogeneity in symptoms [11,14]. Patients vary widely in their disease presentations from asymptomatic to legally blind, even among family members harboring the same mutation [15,16].

The human *OPA1* gene encodes 31 exons and can potentially express eight mRNA isoforms resulting from alternate splicing of exons 4, 4b, and 5b [17,18]. All OPA1 precursor proteins contain an N-terminal mitochondrial targeting sequence (MTS) that allows for the entry into mitochondria where they are further processed into OPA1 protein isoforms [19,20,21,22]. The cleavage of the MTS generates long isoforms (L-OPA1) that are anchored to the inner mitochondrial membrane (IMM) [23,24,25]. L-OPA1 can be further processed by mitochondrial peptidases at several downstream cleavage sites to generate short isoforms (S-OPA1) that are attached to the IMM or distributed in the intermembrane space. The ratio of OPA1 long and short isoforms are dynamically regulated by the mitochondrial inner membrane peptidases OMA1 and YME1L [22], which can respond to stress signals, such as decreased mitochondrial membrane potential and nutrient deprivation [22,26,27].

OPA1 plays critical roles in regulating mitochondrial dynamics, structure, and cellular bioenergetics. Deletion of the *Opa1* gene in mice causes early lethality at embryonic day 9.5 [28]. In contrast, genetically engineered mice with splice site or missense *Opa1* mutations have been shown to mimic human DOA symptoms [29,30,31,32,33,34]. Using Opa1 null mouse embryonic fibroblasts, it was demonstrated that neither long nor short isoforms of OPA1 can function alone [20]. OPA1 promotes mitochondrial fusion along with the mitofusin proteins MFN1 and MFN2 [21,35], and cells carrying *Opa1* mutations or with reduced levels of OPA1 protein show a fragmented mitochondrial network [36]. In addition, OPA1 is required to maintain cristae complexity and structural integrity, thus stabilizing the respiratory chain complexes and controlling cytochrome C release [23,37,38,39,40]. Defects in *Opa1* have been associated with decreases in mitochondrial ATP synthesis and the bioenergetic efficiency of the respiratory complexes [41,42,43].

To date, over 500 pathogenic mutations distributed throughout the *OPA1* gene have been reported (http://www.LOVD.nl/OPA1, accessed on 1 August 2023). Depending on the type and location of the mutation, DOA may occur via dominant negative or haploinsufficiency mechanisms [17,40,44]. Despite the ubiquitous expression of *OPA1*, human cell types other than RGCs are not affected in ~80% of individuals with *OPA1* mutations [45]. Due to the scarcity of human retinal tissues and the rarity of RGCs, which only comprise ~2% of the total human retinal cells [46,47,48], *OPA1*-DOA has not been studied in depth using human RGCs. The high susceptibility of human RGCs to degenerate when OPA1 function is compromised remains not fully understood. Furthermore, recent single-cell transcriptome profiling data have revealed that RGC subtype distribution in primates differs significantly from rodents [49,50]. It is thus necessary to examine human RGCs in order to elucidate the pathological mechanisms responsible for *OPA1* mutation-induced optic nerve degeneration.

Here, we describe the generation of human *OPA1*-mutant pluripotent stem cell (PSC) lines with corresponding isogenic controls as tools for establishing DOA disease models in vitro. Furthermore, we characterized these PSC lines for OPA1 protein expression, mitochondrial morphology, and cellular respiration and energy output. These isogenic PSC lines will be useful tools to investigate how *OPA1* mutations impact PSC-derived human RGCs and facilitate studies of DOA disease mechanisms in vitro.

## 2. Materials and Methods

### 2.1. Human Pluripotent Stem Cell Cultures

Human ESCs and iPSCs were maintained in mTeSR plus medium (Stemcell Technologies, Vancouver, BC, Canada) supplemented with 1% Antibiotic Antimycotic (ThermoFisher Scientific, Canoga Park, CA, USA) on Matrigel (Corning, Corning, NY, USA)-coated plates at 37 °C with 5% CO_2_ as the standard culture condition. PSCs were passaged by dissociating monolayer cells into a single-cell suspension with Accutase (Stemcell Technologies, Vancouver, BC, Canada) and plated in the standard medium containing 10 μM Y-27632 (Stemcell Technologies, Vancouver, BC, Canada) for 24 h. Afterwards, PSCs were returned to the standard medium, which was changed every other day.

### 2.2. CRISPR/Cas9 Gene Editing to Generate OPA1-Mutant ESC Lines

Human UCLA1 (NIH-0058) ESCs with wild-type *OPA1* gene were grown till 80% confluence under the standard condition and dissociated to a single-cell suspension using Accutase (Stemcell Technologies, Vancouver, BC, Canada). RNPs composed of 300 pmol of the synthetic guide RNA, “sgRNA_exon1” (Synthego, Redwood City, CA, USA) (Table S1) and 40 pmol of Cas9 protein (Synthego, Redwood City, CA, USA) were mixed with 5 × 10^5^ UCLA1 ESCs in P3 Primary Cell Nucleofector Solution (Lonza Bioscience, Walkersville, MD, USA) and nucleofected in a Lonza Nucleofector S cuvette using a Lonza 4D-nucleofector X-unit with the CA-137 electroporation program. Nucleofected cells were cultured in mTeSR plus, 1% Antibiotic-Antimycotic, and 10% Clone R (Stemcell Technologies, Vancouver, BC, Canada) for 24 h before returning to the standard medium. To assess the efficiency of CRISPR-Cas9 editing, genomic DNA was extracted using the Purelink genomic DNA mini kit (Invitrogen, Carlsbad, CA, USA) and amplified using Hot Star Taq DNA Polymerase (Qiagen, Hilden, Germany) and primers flanking the double-stranded DNA break site (XJY1349 and XJY1350, Table S2). The resulting PCR products were sequenced using primer XJY1350 (Table S2). The rate of editing in the nucleofected population was assessed using the Inference of CRISPR Edits (ICE) tool (https://ice.synthego.com; V2) [51]. PCR products amplified from UCLA1 ESCs electroporated without any CRISPR reagents were sequenced to generate a control file.

To enable clonal selection, a portion of the nucleofected cells was plated at a density of 1 cell/well in 96-well plates and cultured in mTeSR plus, 1% Antibiotic-Antimycotic, and 10% Clone R. Once the colonies reached ~20 cells, Clone R was removed. Individual colonies were expanded, and their genomic DNA was isolated and PCR-amplified using primers XJY1361 and XJY1362 (Table S2) as described above. The PCR products were sequenced using primer XJY1361 (Table S2) to identify the specific insertion or deletion mutations. Both strands of the genomic DNA around the sgRNA_exon1 of *OPA1* from E10 and D9 mutant ESC lines were sequenced. In addition, all *OPA1*-coding exons of E10 and D9 ESC lines were sequenced to rule out any unintended *OPA1* mutations (Table S2).

### 2.3. CRISPR-HDR Correction of the OPA1 Mutation in 1iDOA iPSC

Prior to nucleofection, 1iDOA iPSCs [52] were dissociated to a single-cell suspension using Accutase (Stemcell Technologies, Vancouver, BC, Canada), and resuspended at a concentration of 25,000 cells/μL in P3 Primary Cell Nucleofector solution (Lonza Bioscience, Walkersville, MD, USA). The solution of RNP composed of 200 pmol of the synthetic sgRNA, “sgRNA_exon19” (Synthego, Redwood City, CA, USA) (Table S1), and 120 pmol of Cas9 protein (ThermoFisher Scientific, Canoga Park, CA, USA) was constituted and incubated at room temperature for 15 min. The single-stranded Alt-R modified donor template (Integrated DNA Technologies, Carolville, IA, USA) (Table S1) was added to the RNP mix to a final concentration of 3 μM along with 5 × 10^5^ 1iDOA iPSCs in P3 Primary Cell Nucleofector solution (Lonza Bioscience, Walkersville, MD, USA). The single-cell suspension was then nucleofected in a Lonza Nucleofector cuvette S using a Lonza 4D-nucleofector X-unit with electroporation program CA-137. Afterwards, cells were cultured as a population in mTeSR plus, 1% Antibiotic-Antimycotic, and 10% Clone R for 24 h before returning to the standard medium.

To facilitate clonal selection, the population of electroporated cells was subsequently plated in Matrigel-coated 96 well plates at a density of 1 cell/well in standard medium containing 10% Clone R. Clone R was removed once colonies reached ~20 cells. Genomic DNA was isolated from expanded colonies using the Purelink genomic DNA mini kit (ThermoFisher Scientific, Canoga Park, CA, USA). The region surrounding the G insertion mutation in exon 19 of *OPA1* was amplified via PCR using primers XJY1366 and XJY1367 (Table S2). PCR products were incubated with or without BstBI (New England Biolabs, Ipswich, MA, USA) and resolved by agarose gel electrophoresis. One iPSC clone (1iDOA-CR) was identified to carry one allele with *BstBI* site and was further verified by DNA sequencing with primers XJY1424-2 and XJY1367 (Table S2). Standard G-band karyotyping was performed by the iPSC core at Cedars Sinai Medical Center (Los Angeles, CA, USA) as previously described [53] to verify that 1iDOA-CR iPSCs displayed a 46, XY normal male karyotype.

### 2.4. Immunofluorescent Labeling, Confocal and Super-Resolution Imaging

PSCs grown on Matrigel-coated plastic coverslips (ThermoFisher Scientific, Canoga Park, CA, USA) were fixed in 4% paraformaldehyde in PBS for 2 min and then incubated in blocking solution (0.1% TritonX-100, 2% donkey serum, 10% FBS in DMEM). Coverslips were sequentially incubated with primary antibodies, followed by incubation with secondary antibodies and 10 μg/mL 4′, 6-diamidino-2-phenylindole (DAPI) diluted in blocking solution (Table S3). All incubations were for one hour at room temperature, and followed by three, 5-min washes in PBS with 0.1% Tween 20. Coverslips were mounted on glass slides and imaged using the Olympus BX61 scanning laser confocal microscope with Plan-APO objectives.

For imaging performed using structured illumination microscopy (SIM), PSCs were grown on #1.5 coverslips (Warner Instruments, Hamden, CT, USA) coated with Matrigel (Corning, Corning, NY, USA). Fixation and immunofluorescent labeling were as described above (Table S3). Coverslips were mounted on glass slides using Vectashield (Vectorlabs, Burlingame, CA, USA) and sealed with CoverGrip (Biotium, Fremont, CA, USA). SIM images were captured using General Electric DeltaVision OMX microscope with a PlanApoN 60×/1.42 NA oil objective (Olympus, Tokyo, Japan). Immersion oil with a refractive index of 1.516 was used. Images were acquired in 3D-SIM mode using a Z-spacing of 0.125 μm and reconstructed using SoftWoRx software 7.2.1 (GE Healthcare Technology, Chicago, IL, USA).

### 2.5. Western Blot

PSCs were washed twice in cold PBS and then incubated with lysis buffer (10 μM HEPES, 10 μM KCL, 0.1% NP40, 1.3 mM MgCl_2_) supplemented with 1× protease and phosphatase inhibitor (Cell Signaling Technology, Danvers, MA, USA) for 2 min at room temperature. Cells were manually dissociated and agitated at 4 °C in lysis buffer for 15 min. Cell extracts were centrifuged at 13,000 rpm at 4 °C for 10 min, after which supernatants were collected. Protein concentration was quantified using the micro-BCA protein assay kit (ThermoFisher Scientific, Canoga Park, CA, USA). 20 μg of protein lysate per sample was loaded on a 4–12% NuPAGE gel (Invitrogen, Carlsbad, CA, USA). Following electrophoresis, the gel was transferred to a PVDF membrane (MilliporeSigma, Temecula, CA, USA) under reducing conditions. The membrane was incubated sequentially with primary and secondary antibodies (Table S3) according to the Near Infrared Western Blot Detection technical guide (LI-COR Biosciences, Lincoln, NE, USA). The Western blots were imaged and quantified using the Odyssey^®^ CLx Imaging System (LI-COR Biosciences, Lincoln, NE, USA). 

### 2.6. Cell Respiration Assays

Human PSCs were dissociated using Accutase and seeded at a density of 10,000 cells/well in a Matrigel-coated Seahorse XF 96-well plate (Agilent, Santa Clara, CA, USA) in 60 μL of standard PSC medium and 10 μM Y-27632. The following day when cells were approximately 80% confluent, the oxygen consumption rate (OCR) and the extracellular acidification rate (ECAR) were measured in parallel in a Seahorse XF96 Extracellular Flux Analyzer (Agilent, Santa Clara, CA, USA). Approximately 1 h prior to analysis, PSC medium was changed to XF assay media (unbuffered DMEM supplemented with 10 mM glucose, 2 mM glutamine, 1 mM pyruvate, and 5 mM HEPES) and the plate was incubated at 37 °C, without CO_2_. Compounds were injected sequentially throughout the assay via injection ports A-D. Final concentrations of injected compounds included: 2 µM oligomycin (Port A), 0.5 µM (Port B) and 0.9 µM (Port C) FCCP, and 2 µM antimycin A and 2 µM rotenone (Port D). Upon assay completion, the plate was washed with PBS and fixed with 4% PFA. Nuclei were stained with 10 ng/mL of Hoechst 33,342 (ThermoFisher, Canoga Park, CA, USA) and counted with an Operetta High-Content Imaging System (PerkinElmer, Tempe, AZ, USA). Rate measurements were normalized to the number of Hoechst-positive nuclei stained before data analysis. Data were analyzed and plotted using the Seahorse Wave Desktop XF software (Agilent, Santa Clara, CA, USA) and exported to Microsoft Excel and GraphPad Prism #9.4.1. ATP production rates were calculated as previously described [54,55].

### 2.7. Statistical Analysis

Seahorse cell respiration data were analyzed using GraphPad Prism #9.4.1 software. Ordinary one-way ANOVA and Tukey’s multiple comparisons tests were used. All error bars are presented as mean value ± SEM. *p* < 0.05 was considered statistically different.

## 3. Results

### 3.1. Generation of Isogenic OPA1-Mutant ESC Lines Using CRISPR-Cas9 Gene Editing

**Figure 1:**
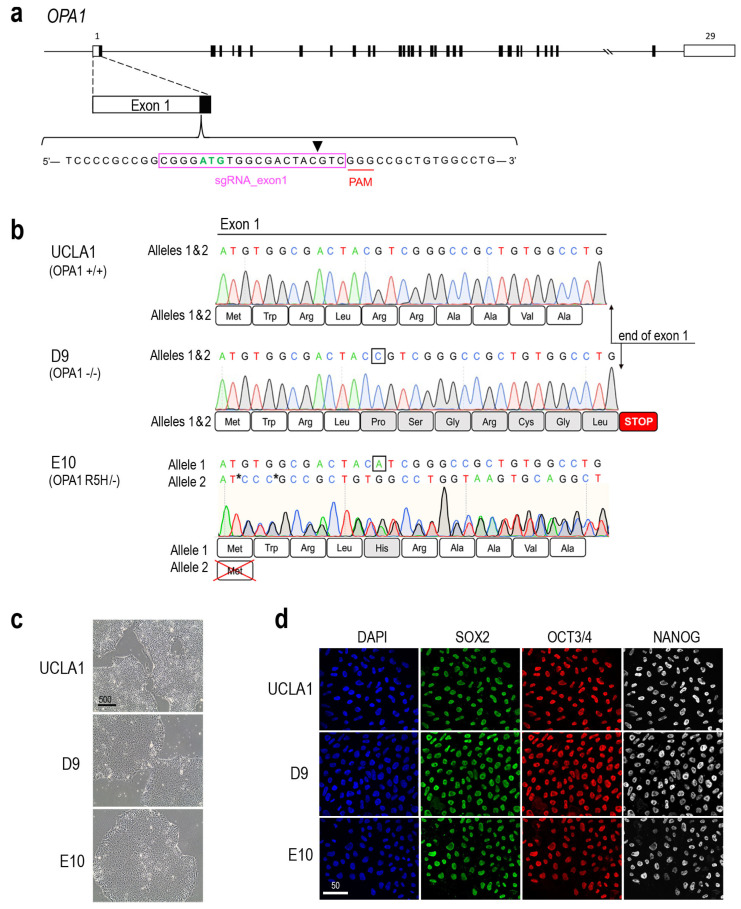
Generation of *OPA1* heterozygous and homozygous mutants isogenic to the WT ESC line UCLA1. (**a**) Schematic drawing of the human *OPA1* gene, which contains 31 exons (exon 1-29, 4b, and 5b). The exons are represented as boxes with protein-coding regions shaded in black. Partial sequence of exon 1 is enlarged to show the ATG translation initiation codon (green), the guide RNA (magenta box), the PAM site (red underline), and the potential CAS9 cleavage site (black arrowhead). (**b**) Alignments of the genomic DNA and predicted protein sequences of the control UCLA1 and the two CRISPR-Cas9 edited OPA1 mutant ESC lines. The control UCLA1 (OPA1+/+) ESC line shows identical DNA sequences for both alleles. The UCLA1-D9 ESC (OPA1−/−) contains a single-base C insertion (boxed) in both alleles, resulting in a frame shift and early stop after 11 amino acids. The UCLA1-E10 ESC (OPA1 R5H/-) has a G > A missense mutation (boxed), resulting in Arg-to-His change (grey shaded box) in allele 1, whereas the allele 2 has a 16-base deletion, which is replaced by a 3-base-pair insertion (3 Cs between the two asterisks), disrupting the ATG start codon. (**c**) Brightfield images show that E10 and D9 display normal pluripotent stem cell morphology comparable to the control UCLA1 ESC line from which they were derived. Scale bar, 500 μm. (**d**) Immunofluorescent labeling of UCLA1, E10, and D9 ESC lines for pluripotent stem cell markers SOX2, OCT3/4, NANOG, and nuclear dye DAPI. Scale bar, 50 μm.

To provide tools for the study of *OPA1*-DOA in vitro, we first generated *OPA1* heterozygous and homozygous mutant ESC lines using CRISPR-Cas9 gene editing. The wild-type (WT) UCLA1 human ESC line was electroporated with ribonucleotide-protein (RNP) complexes consisting of the Cas9 protein and a small guide RNA (sgRNA) targeting exon 1 of the *OPA1* gene (sgRNA_exon1), which was designed to utilize a PAM site near the *OPA1* translation initiation codon to maximize the disruption of protein production from one or both alleles (Figure 1a; Table S1). Following electroporation, the genomic DNA sequence tracing from the edited population were compared to the parental UCLA1 ESCs using the Inference of CRISPR Edits (ICE) tool, which calculates the percentage of insertion or deletion (INDEL) mutations generated by the non-homologous end joining (NHEJ) pathway after Cas9 creates a double-stranded break [51]. The ICE analysis indicated that the RNP-mediated editing was 97% efficient, and 82% of the edits generated were predicted to disrupt OPA1 protein function (not shown).

To identify edited ESC clones that carry either heterozygous or homozygous *OPA1* mutations, the edited UCLA1 cell population was plated as single cells at clonal density. The genomic DNA of expanded ESC clones was analyzed by DNA sequencing of both strands using primers flanking the sgRNA_exon1 cut site (Table S2). The analysis showed that the majority of the ESC clones contained same INDEL mutations on both *OPA1* alleles in the vicinity of the guide RNA targeting site resulting in homozygous *OPA1* mutants. As an example, the ESC clone UCLA1-D9, hereby referred to as D9, contained a single-base C insertion on both alleles (Figure 1b). This mutation caused a frame shift resulting in a premature termination codon after eleven amino acid residuals from the translation start codon (Figure 1b). Since this short peptide abolishes the normal function of OPA1, the ESC clone D9 is predicted as an *OPA1* null mutant (Table 1).

Due to the high efficiency of Cas9-sgRNA_exon1 editing, the resulting *OPA1* heterozygous loss of function clones were rare. We identified one ESC clone UCLA1-E10, here by referred to as E10, harboring compound heterozygous *OPA1* mutations (Figure 1b). Genomic DNA sequencing analysis showed that one allele of E10 carries a G > A missense mutation, which changes the fifth amino acid from arginine to histidine (R5H). The other allele contains an ATG start codon-disrupting deletion resulting in the loss of the normal translation initiation (Table 1). Because protein function prediction software scored the R5H mutation as having a very low likelihood of being pathogenic [56], the ESC clone E10 is thus considered to be similar to an *OPA1* heterozygous mutant and can be used to model DOA disease caused by true haploinsufficiency.

The *OPA1* mutant D9 and E10 ESC lines can be grown and passaged under standard pluripotent stem cell (PSC) culture conditions and show typical ESC morphology compared to the WT parental UCLA1 (Figure 1c). Further, the D9 and E10 ESCs continue to express pluripotent stem cell markers SOX2, OCT3/4, and NANOG as their isogenic parental line UCLA1 (Figure 1d), suggesting that they have retained pluripotent features.

### 3.2. Generation of Isogenic iPSC Lines Using CRISPR-Mediated Homology-Directed Repair

To establish patients’ iPSC-based DOA disease models, we have previously generated DOA patients’ iPSC lines carrying *OPA1* heterozygous mutations [52]. To reduce impacts of genetic backgrounds on DOA disease phenotype analysis, we used CRISPR-Cas9-mediated homology-directed repair (HDR) to correct the *OPA1* gene mutation carried in the iPSC line 1iDOA, generated from a patient with the classic DOA symptoms.

**Figure 2:**
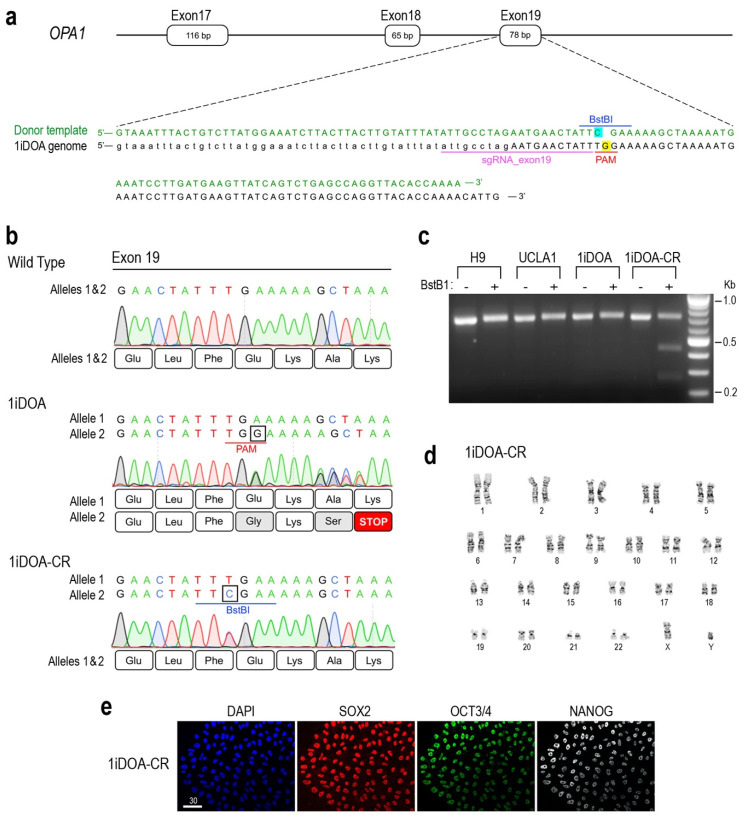
CRISPR-Cas9 mediated correction of the *OPA1* mutation in a DOA iPSC line 1iDOA. (**a**) Schematic drawing depicting the region of the 1iDOA genome carrying a G insertion (yellow highlight) in *OPA1* exon19, the sgRNA_exon19 (underlined in magenta), and the PAM site (underlined in red) absent in the wild-type allele. The 124-nucleotide single-stranded HDR donor template (green) removes the G insertion and introduces a silent T > C mutation (blue highlight), which creates a *BstBI* restriction site (blue overline) on the edited allele. Uppercase letters in the 1iDOA genome (black text) indicate the sequence of *OPA1* exon19 whereas lowercase letters represent intronic sequences. (**b**) Alignments of partial *OPA1* exon19 genomic DNA and predicted protein sequences of the wild-type control, the mutant 1iDOA, and the CRISPR-HDR corrected 1iDOA-CR. DNA sequences for both *OPA1* alleles are shown above of Sanger sequencing profiles. The allele 2 of 1iDOA contains a G insertion (boxed), which leads to a premature stop codon. DNA sequencing confirmed the T > C replacement (boxed) and the *BstBI* site (blue underline) in 1iDOA-CR. Both alleles of 1iDOA-CR encode the wild-type OPA1 protein sequence. Amino acids that differ from the WT protein are shaded in grey. (**c**) Gel image shows the presence of the *BstBI* site in the 1iDOA-CR iPSC line. A 704 bp PCR fragments spanning the area of CRISPR HDR targeting were incubated with or without BstBI and resolved by electrophoresis. Only 1iDOA-CR iPSCs show both the expected 704 bp and two additional bands at 436 and 268 bp, indicating the presence of the novel *BstBI* site. (**d**) The 1iDOA-CR iPSCs displays a normal male karyotype after undergoing CRISPR-Cas9 gene editing. (**e**) Immunofluorescent labeling of 1iDOA-CR iPSCs with pluripotent stem cell markers SOX2, OCT3/4, NANOG, and nuclei dye DAPI. Scale bar, 30 μm.

The iPSC line 1iDOA carried a heterozygous single G insertion in exon19 of the *OPA1* gene (Figure 2a). To carry out CRISPR HDR correction, 1iDOA iPSCs were nucleofected with RNPs consisting of sgRNA_exon19 and Cas9 protein, along with a single-stranded oligodeoxynucleotide (ssODN) repair template. The sgRNA_exon19 was designed to take advantage of the 1iDOA iPSCs’ *OPA1* mutation, which creates a PAM site unique to the mutant allele (Figure 2a), thus allowing for specific targeting by the RNP complex. The ssODN/HDR donor template contained 60 base pair homology arms (Figure 2a) and Alt-R HDR modifications to increase oligo stability and rate of repair [57] (Table S1). In addition to eliminating the G insertion, the HDR donor template also introduced a T > C silent mutation to create a novel *BstBI* restriction site on the corrected allele (Figure 2a,b), which distinguishes the correctly edited clones from other *OPA1* WT lines. The removal of the G insertion mutation from the 1iDOA also eliminated the PAM site, thus preventing further editing of the corrected allele after successful recombination.

Through CRISPR-HDR, we identified a correctly edited clone, 1iDOA-CR (for 1iDOA CRISPR Corrected). Genomic DNA sequencing confirmed that 1iDOA-CR carried the corrected *OPA1* allele eliminating the G insertion and the premature translation stop codon (Figure 2b). In addition, 1iDOA-CR contained the newly created *BstBI* restriction site, which does not exist in 1iDOA mutant iPSC and control wild-type ESC lines (Figure 2c). The HDR corrected 1iDOA-CR iPSCs showed a normal karyotype (Figure 2d) and expressed the pluripotency markers OCT3/4, SOX2, and NANOG (Figure 2e).

### 3.3. OPA1 Protein Expression and Localization in Isogenic PSC Lines

**Figure 3:**
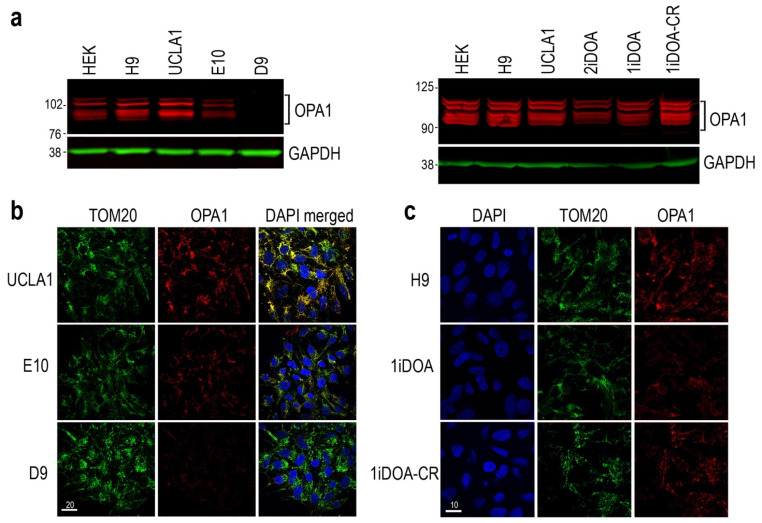
Characterization of OPA1 protein expression in control and mutant PSC lines. (**a**) Western blots showing OPA1 protein expression. The left panel shows the WT control ESC lines H9 and UCLA1, the *OPA1* heterozygous mutant ESC line E10, and the *OPA1* homozygous mutant ESC line D9. The right panel shows the WT control ESC lines H9 and UCLA1, the DOA patients’ iPSC lines 1iDOA and 2iDOA, and the CRISPR corrected iPSC line 1iDOA-CR. All PSC lines except *OPA1* homozygous mutant D9 express OPA1 protein isoforms (~80-100kDa). GAPDH was used as a loading control. Numbers indicate molecular weight marker in kDa. (**b**) Immunofluorescent confocal images show co-labeling for mitochondrial marker TOM20 and OPA1 in parental ESC UCLA1, and isogenic OPA1 mutant ESCs E10 and D9. Scale bar, 20 μm. (**c**) Confocal images show co-labeling for TOM20 and OPA1 in control H9 ESCs, DOA patient-derived 1iDOA, and isogenic 1iDOA-CR iPSCs. Scale bar, 10 μm.

We next investigated the protein expression levels and cellular localization of OPA1 in the established PSC lines. Western blot analysis detected five OPA1 isoforms expressed by HEK 293T cells and by the wild-type H9 ESCs (Figure 3a). Compared to the isogenic parental ESC line UCLA1, the mutant E10 expressed the same isoforms but with reduced OPA1 protein levels, whereas the homozygous mutant D9 showed a loss of OPA1 protein expression (Figure 3a; also see Figure S1). Similarly, Western blot analysis also revealed decreased levels of OPA1 protein expression from DOA patients’ iPSC lines 1iDOA and 2iDOA, which carry distinct heterozygous *OPA1* gene mutations resulting in premature terminations ^53^ (Table 1), compared to the control PSC lines H9 and UCLA1 (Figure 3a; also see Figure S1). Noticeably, compared to the 1iDOA mutant iPSCs, the isogenic 1iDOA-CR iPSC line showed increased OPA1 protein at levels comparable to WT controls, indicating the restoration of OPA1 protein expression after correction of the mutation (Figure 3a).

We next examined the influence of *OPA1* mutations on mitochondria in various PSC lines. Confocal imaging of immunofluorescent labeled mitochondrial outer membrane protein TOM20 showed similar mitochondria presence regardless of *OPA1* genotypes (Figure 3b,c). However, co-labeling for TOM20 and OPA1 revealed that mitochondria of mutant E10 cells contained lower levels of OPA1 signals, whereas the homozygous D9 mutant showed minimal OPA1 labeling compared to the isogenic parental control line UCLA1 (Figure 3b). Consistent with the result of Western blot analysis, the corrected iPSC 1iDOA-CR restored OPA1 labeling in mitochondria compared to the isogenic heterozygous mutant 1iDOA (Figure 3c).

**Figure 4:**
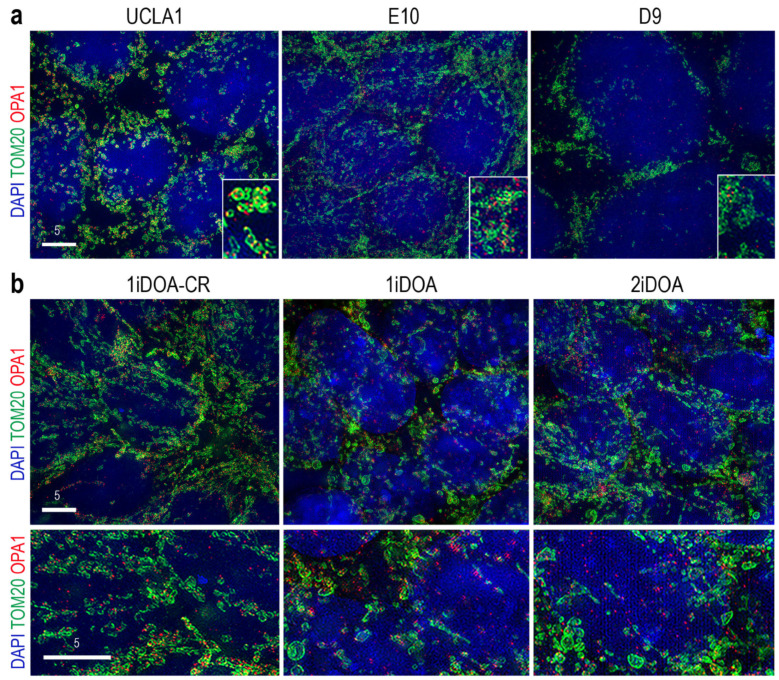
Super-resolution imaging of mitochondria in WT and *OPA1* mutant PSCs. (**a**) Merged SIM images of ESC lines UCLA1, E10, and D9 co-labeled for the mitochondrial marker TOM20, OPA1, and nuclear dye DAPI. The insets are 3x in scale. Scale bars, 5 μm. (**b**) Merged SIM images of iPSC lines 1iDOA-CR, 1iDOA, and 2iDOA co-labeled for the mitochondrial marker TOM20, OPA1, and nuclear dye DAPI. Scale bars, 5 μm.

To assess the impact of OPA1 mutations on mitochondrial morphology in PSCs, we performed high-resolution imaging using structured illumination microscopy (SIM). In the isogenic control ESC line UCLA1, most OPA1 signals were colocalized to mitochondria labeled by TOM20 (Figure 4a). In comparison, SIM imaging revealed reduced TOM20 and OPA1 co-localization in the mutant E10 cells. Furthermore, fragmental mitochondria were detected in the homozygous mutant D9 cells (Figure 4a). SIM imaging also showed a reduced colocalization of TOM20 and OPA1 signals in the heterozygous iPSC line 1iDOA compared with its isogenic control iPSC 1iDOA-CR (Figure 4b).

### 3.4. Impact of OPA1 Mutations on Cellular Respiration and ATP Production

**Figure 5:**
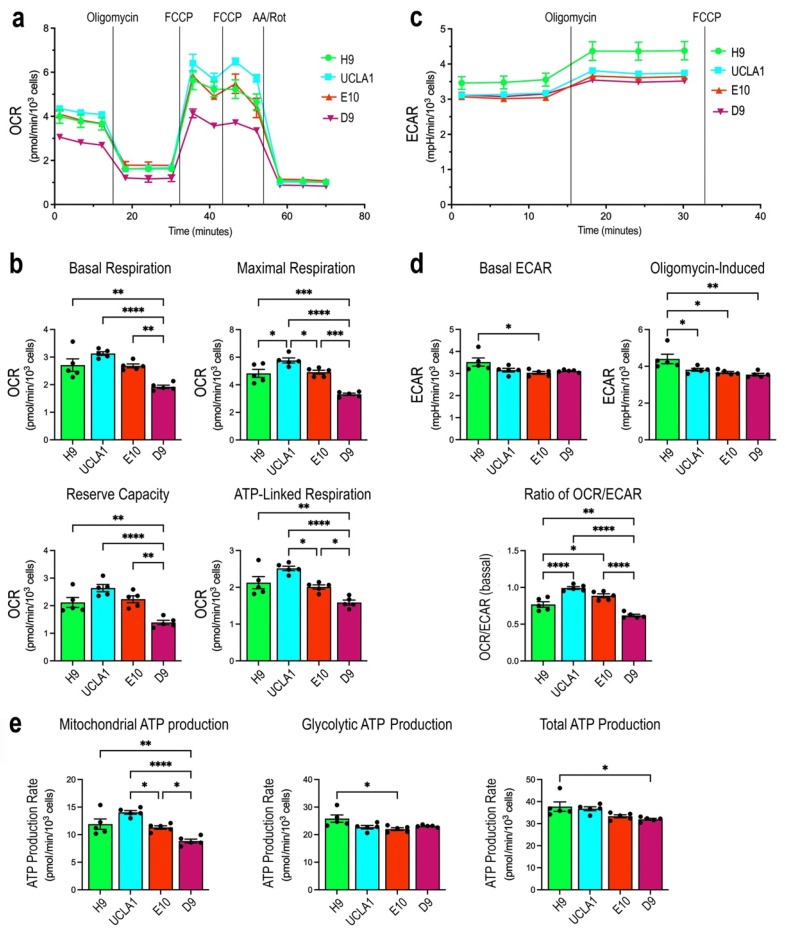
Cellular bioenergetics of normal control and *OPA1* mutant ESCs. The control ESC line H9, the parental ESC UCLA1, and UCLA1-derived *OPA1* mutant ESC lines E10 and D9 were subjected to Seahorse cellular respiration analysis. (**a**) Tracings of OCRs under normal cellular respiration and respiratory chain perturbation conditions are shown. Vertical lines indicate the times of inhibitor applications. (**b**) Bar graphs show quantifications of basal and maximal OCRs, mitochondrial reserve capacities, as well as OCRs linked to ATP production. (**c**) Tracings of ECAR under normal cellular respiration, inhibiting ATP synthase (oligomycin), and uncoupling conditions (FCCP) are shown. (**d**) Bar graphs present quantifications of basal ECAR and ECAR under ATP synthase inhibition. The ratios of OCR/ECAR reflect relative participation of mitochondrial respiration versus cellular glycolysis. (**e**) ATP production rates due to mitochondrial respiration, glycolysis, and total cellular ATP production are presented. *n* = 5 replicates per ESC line. Bar graphs show each *n* as a separate data point, which are presented as mean values +/− SEM. Adjusted *p*-values were obtained from one-way ANOVA and Tukey all-pairs test. * *p* < 0.05, ** *p* < 0.005, *** *p* < 0.0005, **** *p* < 0.0001.

To determine if *OPA1* mutations affect mitochondrial function in various PSC lines, we performed cellular respiration analysis by measuring the oxygen consumption rates (OCR) and the extracellular acidification rates (ECAR). We first examined bioenergetics of the isogenic ESC lines UCLA1, E10, and D9 with glucose and pyruvate as fuels (Figure 5a,c). Compared with the isogenic control UCLA1, the E10 and homozygous D9 mutant ESCs showed reduced basal respiration rates (Figure 5b). After treatments with the ATP synthase inhibitor oligomycin followed by the uncoupler FCCP, E10 and D9 showed deficits in maximal respiration rates, as well as corresponding reduction in mitochondrial reserve capacity and ATP linked respiration (Figure 5b). These significant OCR deficiencies were proportional to copies of *OPA1* mutant alleles. In accordance with the effects of *OPA1* mutations on oxygen consumption, the mitochondrial ATP production rates also showed significant and corresponding decreases in E10 and D9 in comparison to the isogenic control UCLA1 ESCs (Figure 5e). In contrast, the indicator of cellular glycolytic activities ECAR was not significantly affected by *OPA1* mutations (Figure 5d), and the ATP production rates associated with glycolysis did not change among the isogenic ESCs (Figure 5e). As a consequence, the rates of total ATP production in UCLA1, E10, and D9 ESCs did not show statistically significant differences (Figure 5e). The non-isogenic WT ESC line H9 showed a lower mitochondrial ATP production rate but a higher ECAR and glycolytic ATP production (Figure 5e), resulting in a similar total ATP production as UCLA1 (Figure 5e).

**Figure 6:**
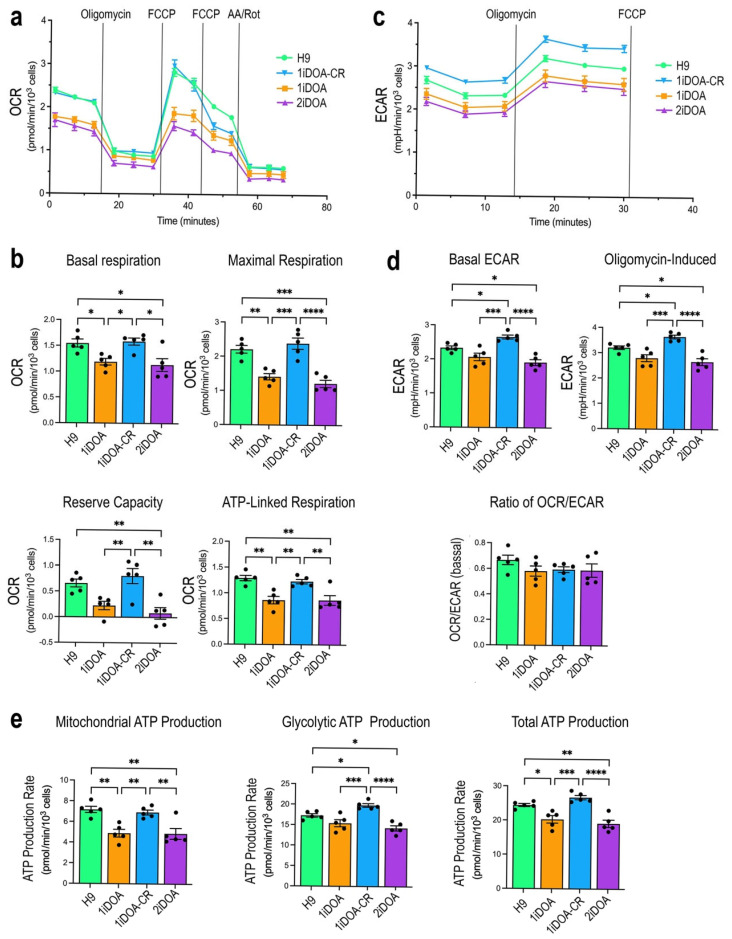
Bioenergetic characterization of control ESC, DOA patients’ iPSC lines, and CRISPR-HDR corrected iPSC line. The control ESC line H9, DOA mutant iPSC lines 1iDOA and 2iDOA, and the CRISPR HDR-corrected iPSC line 1iDOA-CR were subjected to Seahorse cellular respiration analysis. (**a**) Tracings of OCRs under normal cellular respiration and perturbation conditions are shown. Vertical lines indicate times of inhibitor applications. (**b**) Bar graphs present quantifications of basal and maximal OCRs, mitochondrial reserve capacities, as well as OCRs linked to ATP production. (**c**) Tracings of ECAR under normal cellular respiration, inhibiting ATP synthase (oligomycin), or uncoupling (FCCP) conditions are shown. (**d**) Bar graphs present quantifications of basal ECAR and ECAR under ATP synthase-inhibited conditions. The ratios of OCR/ECAR reflect relative participation of mitochondria respiration versus cellular glycolysis. (**e**) ATP production rates due to mitochondrial respiration, glycolysis, and total cellular ATP production are presented. *n* = 5 replicates per cell line. Bar graphs show each *n* as a separate data point, which are presented as mean values +/− SEM. Adjusted *p*-values were obtained from one-way ANOVA and Tukey all-pairs test. * *p* < 0.05, ** *p* < 0.005, *** *p* < 0.0005, **** *p* < 0.0001.

We also performed cellular respiration analysis using *OPA1* mutant iPSC lines 1iDOA and 2iDOA, the CRISPR-corrected iPSC 1iDOA-CR, and the WT ESC line H9. Both *OPA1* heterozygous mutant 1iDOA and 2iDOA iPSCs showed reduced levels of basal, maximal, and ATP-linked respiration, as well as a decreased reserve capacity, compared to the WT control 1iDOA-CR and H9 (Figure 6a,b). In addition, 1iDOA and 2iDOA mutant iPSCs also showed significantly lower levels of ECAR compared to WT control 1iDOA-CR and H9 (Figure 6c,d). In concordance with the reduced OCAR and ECAR, rates of ATP production by mitochondria and glycolysis, as well as the total ATP production rates were significantly reduced in *OPA1* mutant iPSCs compared to WT H9 and 1iDOA-CR (Figure 6e).

## 4. Discussion

Although *OPA1* has been extensively studied in readily accessible cell types, these studies do not solve the conundrum in the field of DOA research: why are RGCs particularly sensitive to *OPA1* mutations? To better address this question and to model OPA1-DOA disease using pluripotent stem-cell-derived human RGCs, we generated *OPA1* heterozygous and homozygous mutant ESC lines from a WT control ESC line and corrected the *OPA1* mutation in a DOA patient iPSC line [52] using CRISPR-Cas9 gene editing technology. Because *OPA1* mutation-driven DOA shows a high degree of heterogeneity and incomplete penetrance, the *OPA1* mutant PSC lines and their isogenic controls can serve as useful research tools for reducing variabilities in DOA disease models in vitro and provide opportunities to investigate *OPA1* deficiency-driven DOA pathogenesis.

Using both *OPA1* ESC and iPSC lines for disease modeling is beneficial, as each confers their own specific advantages. Since the advent of gene editing technology, we are no longer restricted to studying *OPA1* mutations that occur naturally in human patients. Editing a WT ESC line is advantageous in that all derivative lines will be isogenic with the same genetic background. Comparing isogenic ESC lines with or without *OPA1* mutations can increase the reliability in attributing phenotypic differences observed in vitro to a given *OPA1* mutation and avoid issues of incomplete penetrance observed in patient pedigrees. The *OPA1* mutant ESC lines we obtained using CRISPR-Cas9 editing represent the loss of either one or both functional alleles. The E10 ESC line carries a non-expressing null allele and an allele with an R5H missense mutation in the expected mitochondrial targeting sequence. Our results suggest that this mutant line has retained mitochondrial targeting ability and function, thus may resemble a heterozygous scenario. Together, these ESC lines can serve to better understand the requirement for *OPA1* gene dosage during development and pathogenesis.

Conversely, iPSC lines derived from DOA patients with pathogenic *OPA1* variants can be corrected in vitro using gene editing to generate isogenic control iPSC lines. The in vitro phenotypes of DOA patients’ iPSC-derived RGCs can be correlated to patients’ ophthalmological data. Observations from in vitro cell cultures can provide insights into whether the PSC-based disease models recapitulate the DOA disease symptoms and inform what mechanisms underlie these symptoms. Given the heterogeneity of DOA patient population, examining a range of PSC lines with different *OPA1* mutations will be beneficial to fully understand *OPA1*-driven DOA.

Since OPA1 proteins form complexes within mitochondria [25], *OPA1* mutation-triggered DOA disease can occur via dominant negative or haploinsufficiency mechanisms [17,45]. Currently, it remains challenging to correlate the location and type of *OPA1* mutations to the pathogenicity of disease and to predict the mechanism of action for *OPA1* mutant variants [3,8]. We have determined the expression levels of OPA1 protein in the CRISPR-Cas9 editing-generated *OPA1* mutant ESCs. The E10 and the homozygous D9 mutant ESC lines showed reduced and none-detectable OPA1 proteins compared to the WT parental ESC line UCLA1, respectively. The E10 ESC line provides a true loss-of-function scenario, which can be used to represent haploinsufficiency. The two heterozygous DOA patient iPSC lines also showed reduction of OPA1 protein compared to WT control PSCs, while the CRISPR-HDR corrected iPSC restored OPA1 protein expression levels. In *OPA1* mutant PSC lines, we observed the same pattern of OPA1 isoforms [20,58] as their WT counterparts, at relatively equal ratios. Interestingly, mouse embryonic fibroblasts (MEFs) and human embryonic kidney (HEK) 293T cells also express the same OPA1 protein isoforms [58]. These data indicate that these *OPA1* mutations do not alter *OPA1* splicing or post-translational processing in PSCs. Therefore, functional differences detected between WT and *OPA1* mutant PSCs likely result from differences in total OPA1 protein expression levels.

Previous studies have demonstrated that cells lacking *OPA1* expression have highly fragmental mitochondrial networks and reduced cristae complexity [36,40]. Using super-resolution SIM imaging, we also detected differences of mitochondrial morphology between the WT control and the *OPA1* homozygous mutant ESC line UCLA1-D9. However, differences of mitochondrial morphology between WT and *OPA1* heterozygous mutant PSC lines appears subtle in fixed cells by SIM imaging. Unexpectedly, we also observed some cytoplasmic distribution of punctate OPA1 labeling signals with SIM imaging, even in the *OPA1* null ESC lines UCLA1-D9. One possibility is that the OPA1 antibody recognizing the C-terminal portion of OPA1 also binds to other cellular proteins. Alternatively, this could potentially be attributed to the cryptic translation initiation within exon 3 or exon 4 as reported in the NCBI protein database (NP_001341592.1 and NP_001341593.1). These predicted OPA1 proteins lack the mitochondrial targeting sequence encoded by exon 1 and exon 2, therefore may still exist in CRISPR-Cas9 edited ESCs as the guide RNA we used targeted exon 1. However, we have not detected any putative cryptic OPA1 protein isoforms by Western blot analysis. Currently, it remains unclear whether the low levels of OPA1 proteins located in the cytoplasm have any biological function.

It is known that PSCs have high anabolic activities and predominately use glycolysis to provide metabolites for cell proliferation and the maintenance of pluripotency [59]. The mTeSR medium used to culture PSCs contains high glucose (13 mM), which supports glycolysis and anabolism. Our cellular respiration analysis using glucose and pyruvate as fuel sources shows that compared to the WT parental ESC line UCLA1, the isogenic *OPA1* mutant ESCs had significant reduction in their basal, maximal, and ATP-linked respiration. Moreover, these deficits are proportional to the loss of one or both functional *OPA1* alleles (E10 and D9, respectively). However, *OPA1* mutant ESCs retained similar ECARs and glycolytic ATP production as the parental UCLA1 line. This may explain why the OPA1 null mutant D9 ESCs can continue to proliferate, even though they have highly fragmental mitochondria.

Interestingly, DOA patients’ heterozygous mutant iPSC lines not only showed reduced basal, maximal, and ATP-linked respiration rates compared to WT PSCs, they also had reduced basal and oligomycin-induced ECAR, indicating that both oxidative phosphorylation and glycolysis were impaired in DOA patients’ iPSCs. Consequently, in *OPA1* mutant iPSC lines both mitochondrial and glycolytic ATP productions were significantly reduced, causing a decline in total ATP production. It is worth noting that although the DOA patient from whom 2iDOA iPSCs were derived has very mild clinical symptoms [52], the 2iDOA iPSC line displayed similar cellular respiration defects in vitro as 1iDOA, which was derived from a patient with classic DOA symptoms. This observation suggests that *OPA1* mutant phenotypes may more readily manifest under in vitro conditions, and patients’ innate glycolytic activities may play a role in DOA pathogenesis. It would be interesting to assess whether ECAR is consistently reduced in iPSCs of other *OPA1* pathogenic variants, as increasing research has been reported using PSCs to study DOA and other neural degenerative diseases [60,61,62,63,64,65]. Despite the observed differences in ECARs between ESCs and iPSCs, and the reduced cellular respiration capacities of all *OPA1* mutant PSC lines, we did not detect any obvious differences in PSC morphology and growth rates, indicating that under the high glucose culture condition, the cellular metabolic status including the level of cellular ATP is sufficient for PSC maintenance.

Our results demonstrate that cells’ individual genetic backgrounds, in addition to their *OPA1* mutation status, influence their OCR, ECAR, and ATP production in vitro. These differences in basal bioenergetics may contribute to the varied severity of DOA symptoms among individuals with the same *OPA1* mutation.

## 5. Conclusions

We have established human *OPA1* mutant ESC lines by the CRISPR-Cas9 gene editing of a parental control ESC line. In addition, we have developed an isogenic control iPSC line by correcting the pathogenic *OPA1* mutation of a DOA patient’s iPSC using CRISPR-mediated homology-directed repair. Characterization of the isogenic PSCs revealed the impact of different mutations on OPA1 protein expression levels, mitochondrial morphology, and cellular respiration and ATP production. Since *OPA1*-DOA shows incomplete penetrance and varied severity in clinical presentations, these isogenic *OPA1* mutant and control PSCs can serve as useful tools to establish DOA disease models in vitro and evaluate the effects of *OPA1* mutations on PSC-derived human RGCs, thus facilitating disease mechanisms studies and therapeutic treatment development.

## Figures and Tables

**Table 1:** *OPA1* genotypes of pluripotent stem cell lines.

Line	*OPA1* Genotype	*OPA1* Mutation(s)	Effect
UCLA1	+/+	c.473G>A(;)2274T>C p.(Ser158Asn)(;)(Ala758=)	UCLA1 carries two polymorphic mutations that do not affect OPA1 protein function.
			
UCLA1-E10 ^#^	R5H/−	Allele 1: c.14G>A p.(Arg5His) Allele 2: c.3_18delinsCCC p. No translation initiation	Arg5His is a conserved amino acid change. The deletion and insertion mutations result in disrupted ATG start codon.
			
UCLA1-D9 ^#^	−/−	c.[13dup];[13dup] p.[ Arg5ProfsTer8];[Arg5ProfsTer8]	Both alleles have the same single base insertion resulting in premature termination (11 vs. 1015 amino acids)
			
1iDOA	+/−	c.[1948dup];[1948=] p.[Glu650GlyfsTer4];[Glu650=]	One allele has a single base insertion leading to a truncated protein (652 vs. 1015 amino acids)
			
1iDOA-CR	+/+	c.[1947T>C];[1947=] p.(Phe649=)	One allele has a silent mutation that results in a novel BstBI restriction site, but normal proteins.
			
2iDOA	+/−	c.[1417_1418del];[1417_1418=] p.[ Ile473PhefsTer12];[Ile473=]	One allele has a single base insertion leading to a truncated protein (483 vs. 1015 amino acids)
			
H9	+/+	None reported	N/A

The base positions and amino acid positions refer to reference transcript NM_130837.3 and protein sequence NP_570850.2, respectively. +/+: *OPA1* WT; +/−: *OPA1* heterozygous mutant; −/−: *OPA1* homozygous mutant. # ESC lines derived from UCLA1 also contain its polymorphic *OPA1* gene changes at positions 473 and 2274 in exons 4 and 21, respectively.

## Data Availability

All relevant data are included in the manuscript. No new sequencing data beyond verification of CRISPR editing were generated in this study. Any additional information may be obtained by contacting the corresponding author. Requesting of human PSC lines for research by non-profit institutions will undergo an MTA signing process with University of California Los Angeles.

## Supplementary Materials

The following supporting information can be downloaded at: https://www.mdpi.com/article/10.3390/cells14020137/s1, Figure S1: Original Western Blots and Quantification; Table S1: CRISPR-related reagent sequences; Table S2: PCR and Sequencing Primers; Table S3: Antibodies and Dyes.
