# Supplementary material for: Derivation and Characterization of Isogenic OPA1 Mutant and Control Human Pluripotent Stem Cell Lines
Source: Cells. 2025 Jan 17;14(2):137. doi: 10.3390/cells14020137 (PMC11764107; doi:10.3390/cells14020137)
Supplement: Supplementary file 1 [file cells-14-00137-s001.zip › cells-3374401-supplementary.pdf]

Table S1. CRISPR-related reagent sequences

Reagent	Sequence (5'→3')
sgRNA_exon1	CGGGATGTGGCGACTACGTC
sgRNA_exon19	ATTGCCTAGAATGAACTATT
Alt-R HDR donor template (ssODN)	/Alt-R-HDR1/G*T* AAA TTT ACT GTC TTA TGG AAA TCT TAC TTA CTT GTA TTT ATA TTG CCT AGA ATG AAC TAT TCG AAA AAG CTA AAA ATG AAA TCC TTG ATG AAG TTA TCA GTC TGA GCC AGG TTA CAC CAA* A* A/Alt-R-HDR2/

The ssODN contains IDT's modification of phosphorothioate (PS) bonds (indicated by *) and a proprietary end-blocking group on both the 5' and 3' ends (indicated by Alt-R-HDR#). The differences of the ssODN from the 1iDOA genome is highlighted in red: it introduces a silent T>C mutation, removes the G insertion, and creates a *BstBI* site (underlined).

Table S2. PCR and Sequencing Primers

NAME	LOCATION	SEQUENCE (5' - 3')
XJY1336	Exon 1	CACCCGGGATGTGGCGACTACGTC
XJY1337	Exon 1	AAACGACGTAGTCGCCACATCCCG
XJY1338	Exon 7	CACCGTTGAAGTATCAGAGAATCT
XJY1339	Exon 7	AAACAGATTCTCTGATACTTCAAC
XJY1341	Exon 1	GTTGTTTCCGTGACGGACTGAG
XJY1342	Intron 1	CCGAGAGTTAGGAGAAATGTGG
XJY1343	Intron 6	TGTTAAGTGGCCCTGTAATGTG
XJY1344	Exon 7	CTCCATCCTCCAAGCACATTAG
XJY1345	Exon 1	GGGTGAAGTGGTTGTTTCCGTG
XJY1346	Intron 1	GACTCATGGACAGAGCCAAACC
XJY1349	Exon 1	TCTCGGCGCCTGCGTGACCT
XJY1350	Intron 1	TAAGCCCAGCCAGGGCTCCT
XJY1358	Intron 1	TGGTTAGTCACGTATGGGGCTG
XJY1359	Intron 2	TGTGCTCCTTCCACTAGGCCAT
XJY1360	Exon 2	AGCCGATCCTAGTATGAGATAGCG
XJY1361	Exon 1	TGGATTGCTCCAGTCCGTTC
XJY1362	Intron 1	GCAATGTACACATGGCGTGG
XJY1363	Intron 1	ACCAGGATCATAGCCCACATA
XJY1364	Intron 11	GTGAGCGTCTTATCTGAATGG
XJY1365	Intron 13	CCCCTGTGTCTACATTATAGC
XJY1366	Intron 18	CACATAACGTGAACAAGTGT
XJY1367	Intron 19	TACTCAGAATGGAGAACCTG
XJY1368	Intron 1	GACAATAGAGACGGGCCAAG
XJY1369	Intron 2	TTCTTGGCAGGTCTGGGACC
XJY1370	Intron 3	ACAGTTAGTGGCAGCTGTGG
XJY1371	Intron 3	GCCAGTACCACAGCGTAGTG
XJY1372	Intron 4	CCACCAAATTGGTCCAACCAC
XJY1373	Intron 4	GTGGTTGGACCAATTTGGTGG
XJY1374	Intron 4b	CATGACAGGGCAGATGAGAC
XJY1375	Intron 4b	GCACAGGTTATCAGTCATGT
XJY1376	Intron 5	CAGTATGAGAGGTCCTAGGG
XJY1377	Intron 5	GACCAGTAAGATCAACACTG
XJY1378	Intron 5b	CCTATACTACCCACTCCAGA
XJY1379	Intron 5b	GACTAGAATAGCAACAGGGAA
XJY1380	Intron 6	GATGTGGTAGACTCAACTCG
XJY1381	Intron 6	CAATGTGAGTAGCAAGGAA
XJY1382	Intron 7	CACTGGCATACAAGTAAAGC
XJY1383	Intron 7	GTCAGTTGGTTACCTGACC

XJY1384	Intron 8	GCTTAAATTTCACGCATATGG	
XJY1385	Intron 8	GGCTATAAACAGTGGATCCAG	
XJY1386	Intron 9	GGGTCTCTGTCATCCTCTC	
XJY1387	Intron 9	ACCATGTTGACAGCTTCAG	
XJY1388	Intron 11	CCATTCAGATAAGACGCTCAC	
XJY1389	Intron 1	GATTGAGAGGCGTCCAGAAT	
XJY1390	Intron 2	GTCTCCCAGTCTGTCTTCCTT	
XJY1391	Intron 5	CAATCAGTCCTGAGAGAATC	
XJY1392	Intron 5b	GACCCTACAGACAACCTAAT	
XJY1393	Intron 13	GCTATAATGTAGACACAGGG	
XJY1394	Intron 14	CAGTAGAGTCTACTGGGAATG	
XJY1395	Intron 14	GAACTACACATCATTCCGGG	
XJY1396	Intron 16	CAAGGAAGCATGCCAGTATG	
XJY1397	Intron 16	CCTCAGTTACACTAGCCACA	
XJY1398	Intron 17	GTGTACTTCCAAGCACTAGC	
XJY1399	Intron 17	GGTACAACTGCAGAGTCCAT	
XJY1400	Intron 18	GCACATTCATTACCTTCTCAG	
XJY1401	Intron 19	TGAGGATGATGGATGCGTTC	
XJY1402	Intron 20	CTCAGGCCAACATCATACCA	
XJY1403	Intron 20	CCGTGTGAGACCTCTACATC	
XJY1404	Intron 21	AACCAACGCATGTAGTTCCT	
XJY1405	Intron 21	ATTTGGGCCAGGAGAGAATC	
XJY1407	Intron 22	GCCATATCAGTCATGTGGGT	
XJY1408	Intron 23	ACTCAAGCGATTCCTCTGAC	
XJY1409	Intron 23	GTGCTGTGTTCTTTCTTGTG	
XJY1410	Intron 24	GGTCTAGGTCGGTTTCATCC	
XJY1411	Intron 24	CTACCCTGTCTACTCCACA	
XJY1412	Intron 25	CAGGTCAGGGAATAAGGGAC	
XJY1413	Intron 25	CATGGCTCCGTACAGAAAGG	
XJY1414	Intron 26	GCCTCCCAAGTAGCTAGAAC	
XJY1415	Intron 26	GTTGGGAATGGACTCCTAGG	
XJY1416	Intron 27	TAGCTGGGACTATAGGCACC	
XJY1417	Intron 27	TCAGTCTCTCCTCCCACTTC	
XJY1418	Intron 28	GCTCTGTGTTCACCTTTGTTC	
XJY1419	Intron 1	CCAGTATTGAAGCCGGCTCCG	
XJY1420	Intron 1	GTACTGTTACCCTCTCTGATC	
XJY1421	Intron 2	CTAGTCCATGGTAGAGACAC	
XJY1422	Intron 7	GCATTCAGTATTAGCTCATCAG	
XJY1423	Intron 25	GGATAATATGCACGTATCACG	
XJY1424	Intron 18	CCTCCCTTTGGTTATCTCTG	

Table S3. Antibodies and Dyes

	Antibody	Company	Catalog Number	Dilution	Use
Primary	SOX2 (Y-17)	Santa Cruz	sc17320	1:50	IF
	NANOG	abcam	ab21624	1:100	IF
	OCT3/4 (C-10)	Santa Cruz	sc5279	1:50	IF
	OPA1	BD Transduction Laboratories™	612606	1:100; 1:2,000	IF, WB
	GAPDH	Proteintech	10494-I-Ap	1:5,000	WB
	COX IV (3E11)	Cell Signaling	4850S	1:5,000	WB
	TOMM20	abcam	ab186734	1:150	IF, SIM
Secondary	Donkey anti- goat Alexa 488	Invitrogen	A-11055	1:500	IF
	Donkey anti-rabbit Alexa 488	Invitrogen	A-21206	1:500	SIM
	Donkey anti-mouse Alexa 594	Invitrogen	A-21203	1:500	IF
	Donkey anti-rabbit Alexa 647	Invitrogen	A-31573	1:500	IF
	IRDye® 800CW goat anti-rabbit IgG	LI-COR	925-32211	1:20,000	WB
	IRDye® 680RD goat anti-mouse IgG	LI-COR	925-68070	1:20,000	WB
Other	4’, 6-diamidino-2-phenylindole	Sigma	D9542	10 μg/mL final concentration	IF, SIM

IF: Immunofluorescence, WB: Western blot, SIM: Structure illumination microscopy.

Figure S1. Original Western Blots and Quantification.

a

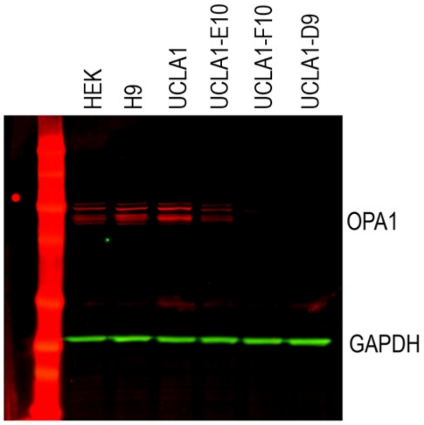

	HEK	H9	UCLA1	E10	F10	D9
OPA1	470	608	725	289	22	9
GAPDH	207	264	229	228	254	322
OPA1/GAPDH	2.27	2.3	3.17	1.27	0.086	0.003

b

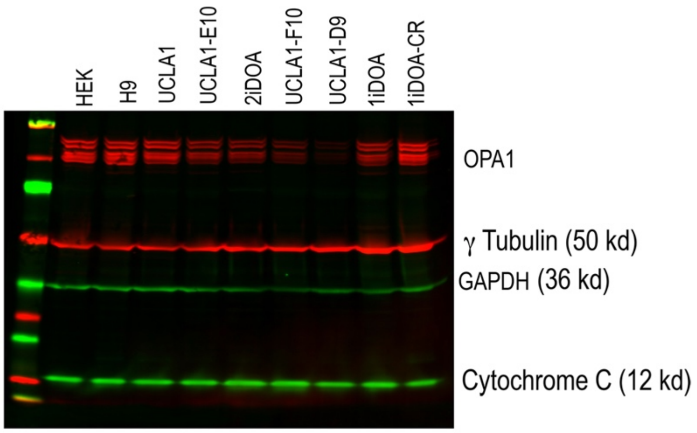

	HEK	H9	UCLA1	E10	2iDOA	F10	D9	1iDOA	1iDOA-CR
OPA1	717	693	558	384	333	216	86	408	617
γTub	237	202	182	236	272	301	377	438	452
COX4	566	541	565	547	614	625	530	585	479
OPA1/γTub	3.04	3.34	3.12	1.63	1.22	0.72	0.23	0.93	1.37
OPA1/COX4	1.27	1.28	1	0.7	0.54	0.34	0.16	0.7	1.29

Figure S1. Original Western Blots and Quantification.

Two original Western blots and quantification of OPA1 proteins are shown. (**a)** OPA1 and loading control GAPDH are quantified, and the ratios of OPA1/GAPDH show reduced OPA1 expression in UCLA1-E10, F10, and D9 compared to the parental UCLA1 ESC line. (**b**) OPA1 and loading controls γTubulin and Cytochrome C (COX4) are quantified. Ratios of OPA1/γTubulin and OPA1/COX4 show reduced expression of UCLA1-E10, F10, and D9 compared to UCLA1. In addition, 1iDOA-CR elevates OPA1 expression in comparison to 1iDOA. Note, F10, a UCLA1 derived ESC line using a different guide RNA, has not been extensively studied.
